# Genome-wide CRISPR synthetic lethality screen identifies a role for the ADP-ribosyltransferase PARP14 in DNA replication dynamics controlled by ATR

**DOI:** 10.1093/nar/gkaa508

**Published:** 2020-06-15

**Authors:** Ashna Dhoonmoon, Emily M Schleicher, Kristen E Clements, Claudia M Nicolae, George-Lucian Moldovan

**Affiliations:** Department of Biochemistry and Molecular Biology, The Pennsylvania State University College of Medicine, Hershey, PA 17033, USA; Department of Biochemistry and Molecular Biology, The Pennsylvania State University College of Medicine, Hershey, PA 17033, USA; Department of Biochemistry and Molecular Biology, The Pennsylvania State University College of Medicine, Hershey, PA 17033, USA; Department of Biochemistry and Molecular Biology, The Pennsylvania State University College of Medicine, Hershey, PA 17033, USA; Department of Biochemistry and Molecular Biology, The Pennsylvania State University College of Medicine, Hershey, PA 17033, USA

## Abstract

The DNA damage response is essential to maintain genomic stability, suppress replication stress, and protect against carcinogenesis. The ATR-CHK1 pathway is an essential component of this response, which regulates cell cycle progression in the face of replication stress. PARP14 is an ADP-ribosyltransferase with multiple roles in transcription, signaling, and DNA repair. To understand the biological functions of PARP14, we catalogued the genetic components that impact cellular viability upon loss of PARP14 by performing an unbiased, comprehensive, genome-wide CRISPR knockout genetic screen in PARP14-deficient cells. We uncovered the ATR-CHK1 pathway as essential for viability of PARP14-deficient cells, and identified regulation of DNA replication dynamics as an important mechanistic contributor to the synthetic lethality observed. Our work shows that PARP14 is an important modulator of the response to ATR-CHK1 pathway inhibitors.

## INTRODUCTION

The DNA damage response (DDR) machinery is essential to maintain genomic stability, ensure cellular proliferation and protect against carcinogenesis (1). The complex mechanisms employed by the DDR participate not only in repairing DNA damage, but also in attenuating replication stress (2,3). Arrest of the DNA polymerases at sites of replication blockades can result in collapse of the replication machinery and genomic instability. A crucial component of the DDR is the ataxia telangiectasia and Rad3-related (ATR) protein kinase, which is activated by single stranded DNA induced upon replication stress. This leads to downstream phosphorylation of Chk1, which induces a broad cellular response resulting in stabilization of the replication fork, suppression of origin firing, and cell cycle arrest (4–6). ATR and CHK1 inhibitors are currently being investigated as anti-cancer drugs, with multiple clinical trials under way (7,8).

ADP-ribosylation is a prominent post-translational modification which regulates transcription, signal transduction, and DNA repair (9–12). PARP1 is the best characterized member of this family. In particular, PARP1 has emerged as potent target for treatment of tumors with deficiency in the BRCA pathway of homologous recombination (HR) DNA repair, as unrepairable DNA damage results in death of HR-deficient cells treated with PARP1 inhibitors such as olaparib (13,14). This synthetic lethal interaction between PARP1 and the BRCA pathway has been effectively employed for clinical treatment of ovarian, breast and pancreatic cancer (15).

Besides PARP1, the PARP family comprises 16 other members, with various and less understood functions (16). PARP14 (also known as ARTD8) has been associated with multiple cellular processes, however mechanistic details are generally sparse (17). PARP14 has been shown to be involved in regulation of multiple signal transduction pathways including NFκB (18–20), and JNK (21,22). Moreover, PARP14 has been described as a transcriptional co-activator regulating the macrophage-specific transcriptional program (23–25). More recently, it has been shown that PARP14 interacts with multiple RNA regulatory proteins and may play a role in regulating RNA stability (23,26). PARP14 catalytic inhibitors are currently being developed and targeting PARP14 has been proposed as a possible therapeutic approach for multiple cancer types (18,21,22,27,28).

We previously showed that PARP14 is essential for genomic stability by promoting HR and alleviating replication stress (29). Mechanistically, we showed that PARP14 regulates the association of the RAD51 recombinase, an essential HR factor, with damaged DNA. These findings further indicate that PARP14 may impact the tumor response to treatment with genotoxic drugs.

With the advent of the genomics era, and the concomitant development of numerous novel drug targets, it has become clear that identification of the genetic background that confers maximum drug sensitivity is paramount for advancing cancer therapy. Genome-wide genetic screens in human cells have proven invaluable tools to comprehensively and unbiasedly evaluate pharmacogenetic interactions (30–32). Moreover, such screens can provide invaluable insights into functions and mechanisms of human genes. Here, we describe a genome-wide CRISPR-based knockout screen designed to identify synthetic lethality interactions of PARP14. We show that the ATR-CHK1 pathway is essential for viability of PARP14-deficient cells, and identify regulation of replication dynamics as an important mechanistic contributor to the synthetic lethality observed. Our work shows that PARP14 is an important modulator of the response to ATR–CHK1 pathway inhibitors.

## MATERIALS AND METHODS

### Cell culture

Human 8988T and U2OS cells were grown in Dulbecco's modified Eagle's medium (DMEM). DLD-1 cells were grown in Roswell Park memorial Institute (RPMI) 1640 medium. DMEM and RPMI were both supplemented with 10% FBS and penicillin/streptomycin. To generate the 8988T PARP14-knockout cells, the commercially available PARP14 CRISPR/Cas9 KO plasmid was used (Santa Cruz Biotechnology sc-402812). Transfected cells were FACS-sorted into 96-well plates using a BD FACSAria II instrument. Resulting colonies were screened by Western blot. To re-express exogenous PARP14 in the knockout cell lines, cells were infected with the lentiviral construct pLV-Puro-SV40>Flag/hPARP14 (Cyagen) was used, constitutively expressing Flag-tagged PARP14 under the control of the SV40 promoter.

Gene knockdown was performed using Lipofectamine RNAiMAX transfection reagent. AllStars Negative Control siRNA (Qiagen 1027281) was used as control. The following oligonucleotide sequences (Stealth siRNA, ThermoFisher) were used: PARP14: AGGCCGACTGTGACCAGATAGTGAA; DNA2: TTAGAATGCAGGCAACTGTATCCTT; MRE11: CATTACATACCTGCCTCGAGTTATT; TOPBP1: Silencer Select ID s2183; CHK1: Silencer Select ID s504.

Denatured whole cell extracts were prepared by boiling cells in 100 mM Tris, 4% SDS, 0.5M β-mercaptoethanol. Antibodies used for western blot were: PAPR14 (Santa Cruz Biotechnology sc-377150); Chk1 (Cell signaling Technology 2360); DNA2 (Abcam ab96488); MRE11 (Santa Cruz Biotechnology sc-135992); TOPBP1 (Novus NB100-217); GAPDH (Santa Cruz Biotechnology sc-47724); γH2AX (Abcam ab-2893). The chemical inhibitors used in this study were obtained from Selleck Chemicals: rabusertib (CHK1i); VE822 (ATRi); olaparib (PARP1i).

### CRISPR screens

For CRISPR knockout screens, the Brunello Human CRISPR knockout pooled lentiviral library (Addgene 73179) was used (33). This library targets 19 114 genes with 76 411 guide RNA (gRNA) sequences. 100 million 8988T (wildype and PARP14^KO6^) cells were infected with this library at a multiplicity of infection (MOI) of 0.4 to achieve 500× coverage and selected for 4 days with 1.25 μg/ml puromycin. The same lentiviral preparation of the library was used to infect both cell lines, to ensure similar guide representation. For each condition, 20 million cells freshly infected with the library (to maintain 250× coverage) were seeded and allowed to grow for two weeks. Genomic DNA was isolated using the DNeasy Blood and Tissue Kit (Qiagen 69504) per the manufacturer's instructions. The gRNA sequences were amplified using PCR primers with Illumina adapters. Genomic DNA from 20 million cells (250-fold library coverage) was used as template for PCR. The PCR reaction contained 10 μg of gDNA, with 20 μl 5× HiFi Reaction Buffer, 4 μl of P5 primer, 4 μl of P7 primer, 3 μl of Radiant HiFi Ultra Polymerase (Stellar Scientific), and water. The P5 and P7 primers used were determined using the user guide provided with the CRISPR libraries (https://media.addgene.org/cms/filer_public/61/16/611619f4-0926-4a07-b5c7-e286a8ecf7f5/broadgpp-sequencing-protocol.pdf). The purified PCR product was sequenced with Illumina HiSeq 2500 single read for 50 cycles. The percentage of undetected guides was 0.5% for wildtype cells and 0.4% for PARP14-knockout cells, respectively. The skew ratio of top 10% to bottom 10% guides was 7.3 for wildtype cells and 10.7 for PARP14-knockout cells, respectively. Both parameters are within the recommended range, indicating an appropriate library coverage.

For bioinformatic analysis of the screen results (Supplementary Figure S1), the custom python script provided (count_spacers.py) (34) was used to calculate gRNA representation. The difference between the number of guides present in the PARP14-knockout condition compared to the wildtype condition was determined. Specifically, one read count was added to each gRNA, and then the reads from the PARP14-knockout condition were normalized to the wildtype condition. The values obtained were then used as input in the Redundant siRNA Activity (RSA) algorithm (35), which takes into consideration the ranking of each individual gRNA targeting a gene to rank all genes. For RSA, the Bonferroni option was used and guides that were at least 2-fold enriched in the PARP14-knockout condition compared to the wildtype condition were considered hits. The *P*-values are determined by the RSA algorithm for the genes that are most enriched in the PARP14-knockout condition compared to the wildtype condition. Separately from the RSA analyses, we also analyzed the screen results using MAGeCK, which takes into consideration raw gRNA read counts to test if individual guides vary significantly between the conditions (36,37). The MAGeCK software and instructions on running it were obtained from https://sourceforge.net/p/mageck/wiki/libraries/. Finally, analyses of the Gene Ontology pathways enriched among the top hits was performed using DAVID (38,39).

### Functional cellular assays

For clonogenic survival assays, 500 cells were seeded per well in 6-well plates and treated with siRNA or drug as indicated. Media was changed after 3 days and cells were allowed to grow for 10–14 days. Colonies formed were then washed with PBS, fixed with a solution of 10% methanol + 10% acetic acid and stained with crystal violet (2%, aqua solutions). For crystal violet imaging, 50 000 cells were seeded per well in 12-well plates and treated with siRNA or drug as indicated. Staining was performed 3 days later as described above. To assess cellular proliferation, a luminescent ATP-based assay was performed using the CellTiterGlo reagent (Promega G7572) as per manufacturer's instructions. Following treatment with siRNA, 1500 cells were seeded per well (day 0) and plates were read daily for 5 days. For drug sensitivity, 1500 cells were seeded per well in 96-well plated and treated with the indicated drug doses. Plates were read 3 days later. For apoptosis assays, cells were treated with siRNA for 2 days, followed by media change. Cells were prepared for flow cytometry two days after media change using the FITC Annexin V kit (Biolegend, 640906). Quantification was performed using a BD FACSCanto 10 flow cytometer. For quantification of G1-S cells positive for histone H3 phosphorylated at Ser10, the Click-iT Plus EdU Alexa Fluor 488 Flow Cytometry Assay Kit (ThermoFisher) was used to measure cell cycle distribution, according to the manufacturer's instruction. Concomitantly, cells were stained with the Phospho-Histone H3 (Ser10) Alexa Fluor 594 conjugated antibody. Cells were subsequently analyzed by flow cytometry.

### DNA fiber assays

For the experiments with gene knockdown, cells were treated with siRNA for 2 days, then incubated with 100 μM IdU for 30 min, washed with PBS and incubated with 100 μM CldU. For the experiments with drug treatment, cells were incubated with 100 μM IdU for 30 min, washed with PBS, and incubated within the drugs and/or CldU as indicated. Next, cells were collected and processed using the the FiberPrep kit (Genomic Vision EXT-001) according to the manufacturer's instructions. DNA molecules were stretched onto coverslips (Genomic Vision COV-002-RUO) using the FiberComb Molecular Combing instrument (Genomic Vision MCS-001). Slides were stained with antibodies detecting CldU (Abcam 6236), IdU (BD 347580) and DNA (Millipore Sigma MAD3034). Slides were then incubated with secondary Cy3, Cy5 or BV480-conjugated antibodies (Abcam 6946, Abcam 6565 and BD Biosciences 564879). Finally, the cells were mounted onto coverslips and imaged using a confocal microscope (Leica SP5).

### Statistical analyses

For CellTiter-Glo cellular proliferation assays, the two-way ANOVA statistical test was used. This test was also used for drug sensitivity clonogenic assay. For clonogenic survival assays upon gene knockdown by siRNA, as well as for the Annexin V assay, the *t*-test (two-tailed, unequal variance unless indicated) was used. For the DNA fiber assay, the Mann–Whitney statistical test was performed. Statistical significance is indicated for each graph (ns = not significant, for *P*> 0.05; * for *P*≤ 0.05; ** for *P*≤ 0.01; *** for *P*≤ 0.001, **** for *P*≤ 0.0001).

## RESULTS

### Genome-wide CRISPR screen identifies PARP14 synthetic lethal candidates

**Figure 1.**
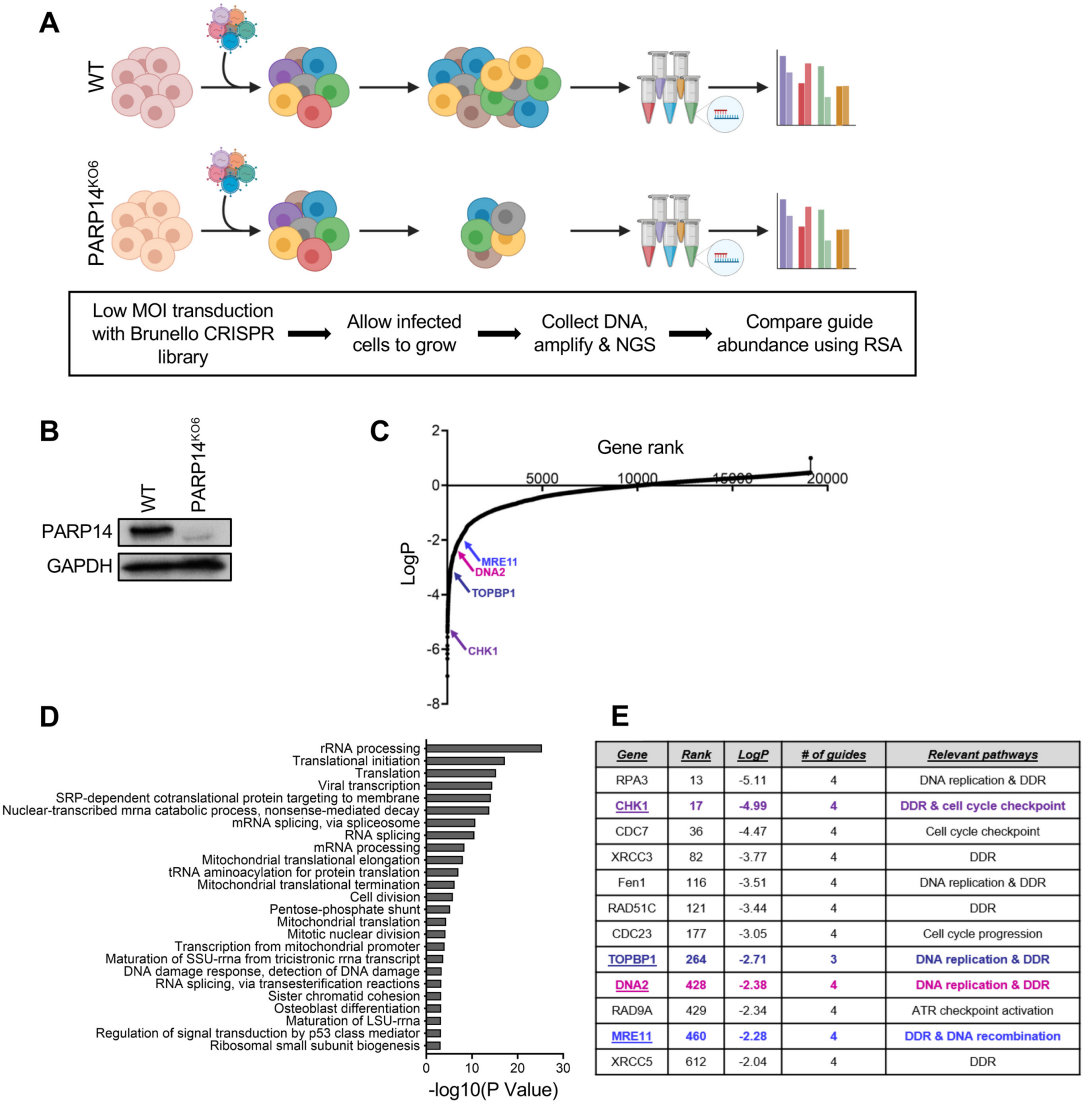
Genome-wide CRISPR knockout screen identified genes essential for viability of PARP14-knockout cells. (**A**) Schematic representation of CRISPR knockout screen. 8988T wild type (WT) and PARP14-knockout (PARP14^KO6^) were infected with the Brunello CRISPR knockout library and allowed to grow for two weeks. Genomic DNA was then extracted from both groups of cells and gRNAs were identified using Illumina sequencing. (**B**) Western blot showing loss of PARP14 protein in the 8988T PARP14^KO6^ cells. (**C**) Scatterplot ranking the genes targeted by library according to *P*-values is shown. RSA analysis was used to obtain gene ranking. (**D**) Pathway analysis showing the biological processes that were significantly enriched in the top 500 hits (genes lost in the PARP14^KO6^ cells compared to wildtype, ranked using RSA). The top 25 Gene Ontology (GO) terms are shown. A similar analysis of the top 500 hits ranked by MAGeCK is shown in Supplementary Figure S2A. (**E**) Multiple DNA damage response (DDR) genes were among the top hits ranked using RSA. The highlighted candidates, namely CHK1, DNA2, TOPBP1 and MRE11, were validated in this study. A comparison of the ranking of these genes by RSA and MAGeCK is shown in Supplementary Figure S2B.

In order to identify genes which are essential for cellular viability in the absence of PARP14, we performed a genome-wide synthetic lethality CRISPR knockout screen in 8988T pancreatic cancer cells (Figure 1A). We chose the 8988T cell line as we previously (29) used it for PARP14-knockdown studies and found no significant impact on proliferation or cell cycle progression. In addition, 8988T cells are sturdy, amenable to transfection and viral transduction, and have good proliferation rates, making 8988T an appropriate cell line for the experimental setup employed here. First, we obtained PARP14-knockout 8988T cells by CRISPR/Cas9-mediated genome editing (Figure 1B). Out of the several PARP14-knockout clones obtained, the KO6 clone (PARP14^KO6^) was used for the synthetic lethality screen. Wildtype and PARP14^KO6^ 8988T cells were infected with the Brunello human CRISPR knockout lentiviral-based library. This library targets 19 114 genes with a total of 76 441 unique gRNA sequences, thus on average covering each gene with four different gRNAs (33). To maintain 250-fold library coverage, 20 million library-infected cells were allowed to grow for two weeks. Cells were then collected, and genomic DNA was extracted. The gRNA region was amplified by PCR and identified by Illumina sequencing (Figure 1A).

We next employed, in parallel, two different computational algorithms, namely RSA and MAGeCK (35–37) to generate a ranked list of genes that were lost in PARP14-knockout compared to the wildtype control condition (Figure 1C; Supplementary Tables S1 and S2). There was a high degree of similarity between the gene rankings generated by the two algorithms (Supplementary Tables S1 and S2). Biological pathway analysis of the top 500 hits revealed RNA-related processes as the most commonly enriched in synthetic lethal interactions with PARP14 loss (Figure 1D, Supplementary Figure S2A, Table S3), perhaps in line with previously proposed roles for PARP14 in regulating RNA stability (23,26). Another biological process highly represented on the pathway analysis and previously associated with PARP14 was regulation of mitochondrial activity (21,27). However, cell division, chromosome biology and DNA replication and repair also feature prominently on the list (Figure 1D, E). In particular, among the top hits were multiple components of the ATR pathway, including CHK1, TOPBP1, MRE11, RPA3 and RAD9A (Figure 1E, Supplementary Figure S2B). Since we previously described a role for PARP14 in DNA repair (29), we decided to focus on the experimental validation of this class of hits.

### Loss of CHK1 or DNA2 reduces proliferation of PARP14-deficient cells

**Figure 2.**
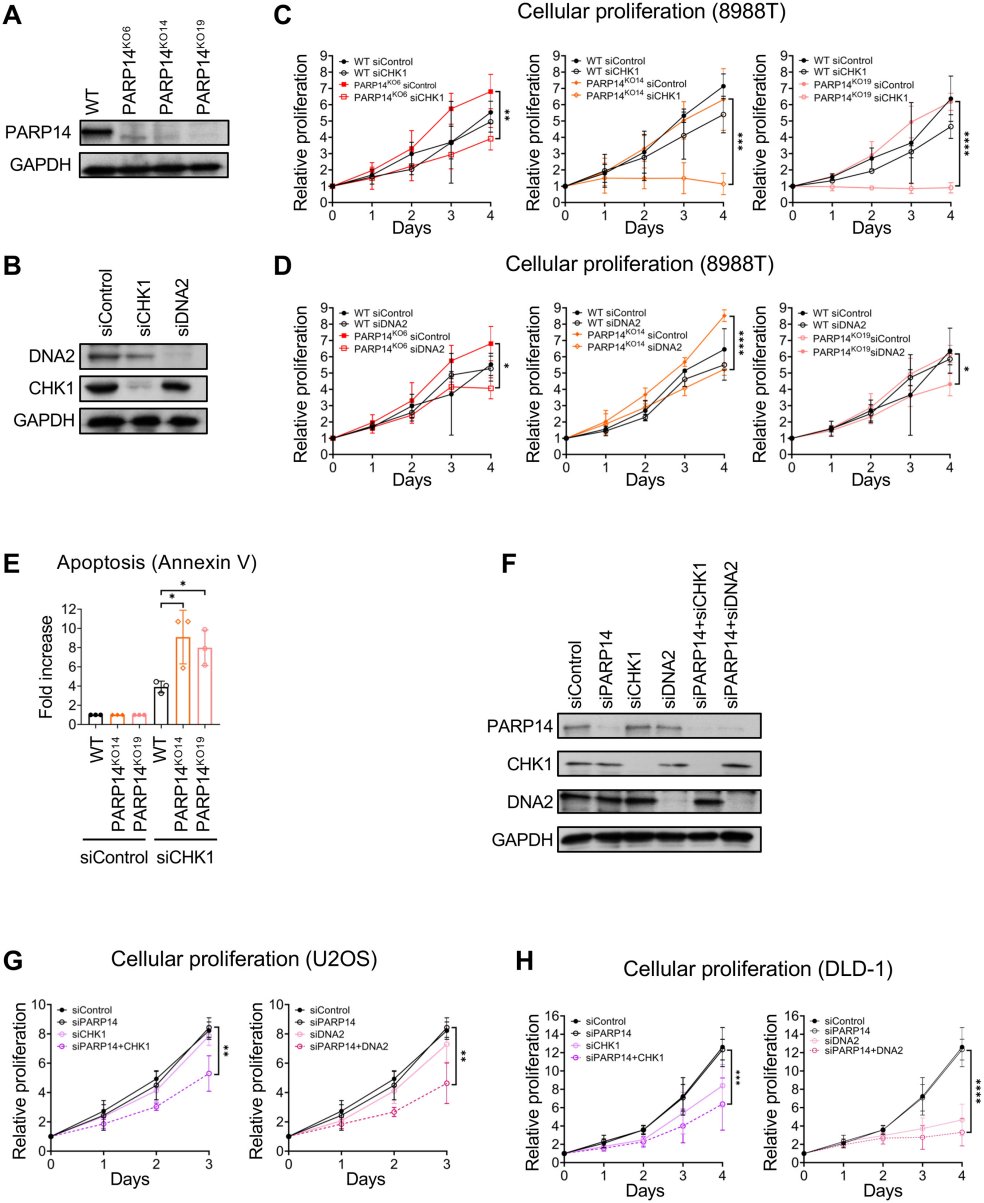
Knockdown of CHK1 or DNA2 reduces proliferation of PARP14-deficient cells. (**A**) Western blot showing the loss of PARP14 protein in multiple 8988T knockout clones. (**B**) Western blot showing efficient siRNA-mediated downregulation of CHK1 and DNA2 in 8988T cells. (**C**) Cellular proliferation assay showing that CHK1 knockdown reduced proliferation of all three PARP14-knockout 8988T clones compared to control. The average of three experiments is presented, with standard deviations shown as error bars. Asterisks indicate statistical significance. (**D**) Cellular proliferation assay showing that DNA2 knockdown reduced proliferation of all three PARP14-knockout 8988T clones compared to control. The average of three experiments is presented, with standard deviations shown as error bars. Asterisks indicate statistical significance. (**E**) Annexin V assays demonstrating increased apoptosis in multiple PARP14-knockout 8988T clones upon CHK1 knockdown. The average of three experiments is presented, with standard deviations shown as error bars. Asterisks indicate statistical significance. (**F**) Western blot showing efficient siRNA-mediated co-depletion of PARP14 and CHK1 or DNA2 in DLD-1 cells. (**G, H**) Co-depletion of CHK1 or DNA2 reduces proliferation of PARP14-knockdown U2OS (**G**) and DLD-1 (**H**) cells. The average of three experiments is presented for U2OS cells and average of four experiments is presented for DLD-1 cells, with standard deviations shown as error bars. Asterisks indicate statistical significance.

For screen validation, as a proof of concept we first picked two of the functionally relevant top candidates, namely CHK1 and DNA2. Both CHK1 and DNA2 are key players in DNA damage repair and represent potential therapeutic targets for cancer therapy (40–42). To validate these candidates, we used both the original 8988T PARP14^KO6^ cell line in which the screen was performed, as well as two additional PARP14-knockout 8988T clones, namely PARP14^KO14^ and PARP14^KO19^ (Figure 2A). We employed siRNA to knockdown the candidates in these cells (Figure 2B), and measured their proliferation over four days using the CellTiterGlo ATP-based luminescence assay. While PARP14 knockout by itself did not affect proliferation or cell cycle progression, knockdown of CHK1 or DNA2 led to impaired cellular proliferation in all three 8988T PARP14-knockout clones compared to wildtype cells (Figure 2C, D; Supplementary Figure S3). We also measured apoptosis using Annexin V flow cytometry upon CHK1 depletion in PARP14-knockout cells. Treatment of multiple PARP14-knockout 8988T clones with siRNA targeting CHK1 significantly increased apoptosis compared to control cells (Figure 2E).

In order to rule out any cell line-specific effects, we next sought to validate CHK1 and DNA2 in two additional cell lines, namely U2OS (human osteosarcoma) and DLD-1 (colorectal adenocarcinoma). For these two cell lines, we performed co-depletion of PARP14 and either CHK1 or DNA2 using siRNA. Western blot experiments indicated that co-depletion was efficient (Figure 2F). In line with the findings in 8988T cells, loss of both PARP14 and CHK1 or DNA2 reduced cellular proliferation in U2OS (Figure 2G) and DLD-1 (Figure 2H) cell lines. These results indicate that CHK1 and DNA2 are essential for proliferation of PARP14-deficient cells.

**Figure 3.**
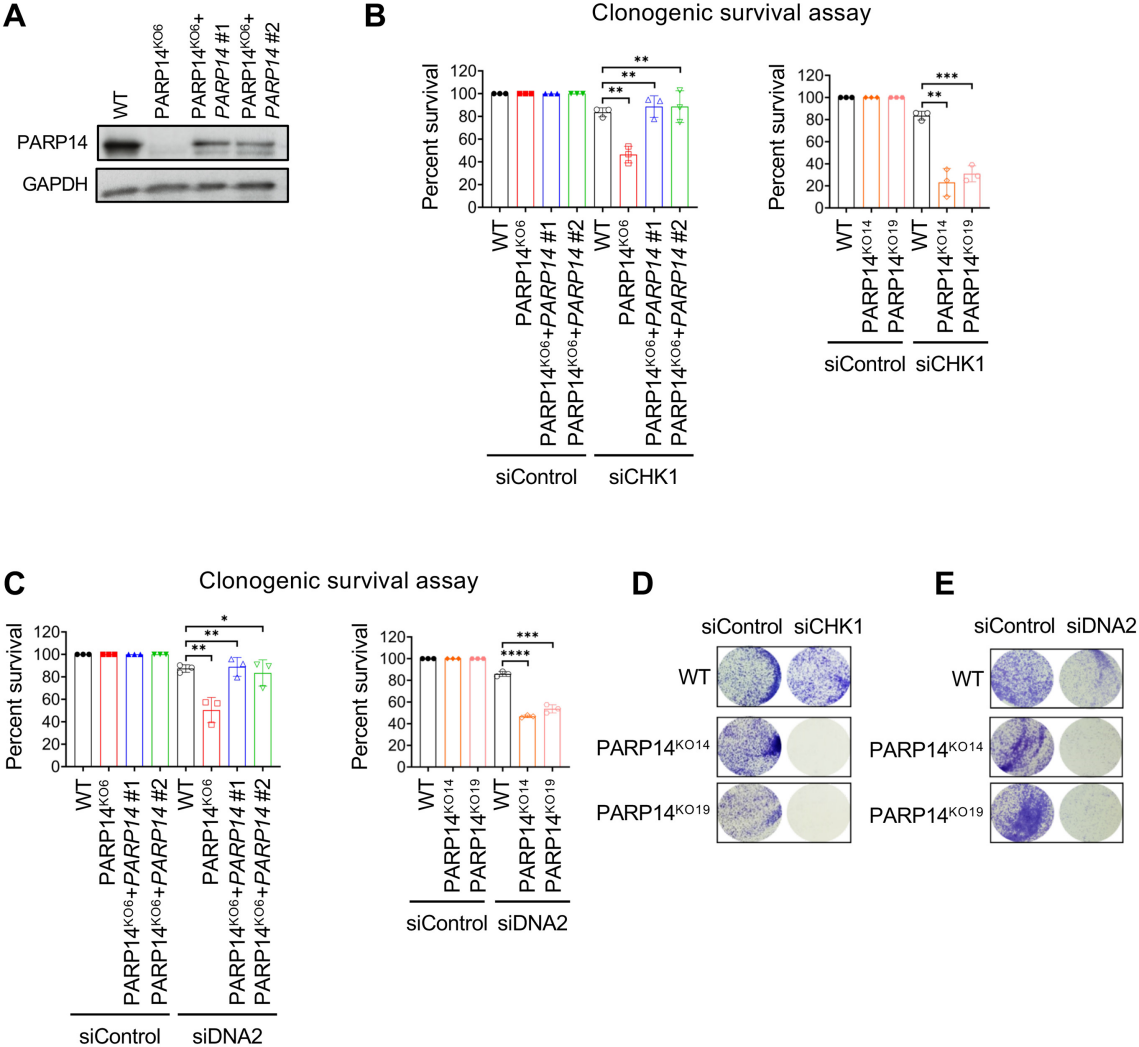
Reduced viability of PARP14-knockout cells upon depletion of CHK1 or DNA2. (**A**) Western blot showing re-expression of PARP14 in the 8988T knockout cells corrected with *PARP14* cDNA. Two different clones were obtained and are investigated here. (B, C) Clonogenic survival assays showing reduced survival of PARP14-knockout 8988T cells upon CHK1 (**B**) or DNA2 (**C**) knockdown. All three PARP14-knockout clones were investigated and showed similar phenotypes. Re-expression of exogenous PARP14 in the knockout cells rescued the survival. The average of three experiments is presented, with standard deviations shown as error bars. Asterisks indicate statistical significance. (D, E) Representative images of crystal violet staining showing the reduced viability of PARP14-knockout 8988T cells upon depletion of CHK1 (**D**) or DNA2 (**E**). Two different knockout clones show the same phenotype.

Next, we tested how long-term viability is affected when the top candidates are depleted in the PARP14-knockout cells. To this end, we performed clonogenic survival assays in 8988T cells. In all three knockout clones, siRNA-mediated depletion of CHK1 resulted in severely impaired colony formation (Figure 3A, B). To rule out off-target effects of the CRISPR gene editing system employed, we corrected the PARP14^KO6^ clone by exogenous, constitutive re-expression of *PARP14* cDNA. Two separate re-expression clones (#1 and #2) were obtained (Figure 3A). Re-expression of PARP14 in the KO6 clone restored the clonogenic survival upon CHK1 depletion to wildtype levels (Figure 3B). Similar to CHK1, depletion of DNA2 in all three 8988T PARP14-knockout clones also resulted in reduced clonogenic survival, which was rescued upon re-expression of PARP14 cDNA in the PARP14^KO6^ clone (Figure 3C). Moreover, the synthetic lethality interaction between PARP14 and CHK1 or DNA2 was further validated by crystal violet staining of plates seeded at high density with PARP14-knockout cells treated with siRNA targeting these factors (Figure 3D, E). These findings confirm that PARP14 is synthetic lethal with CHK1 and DNA2, thus validating our genome-wide synthetic lethality screen.

### Synthetic lethality between PARP14 and ATR pathway components

**Figure 4.**
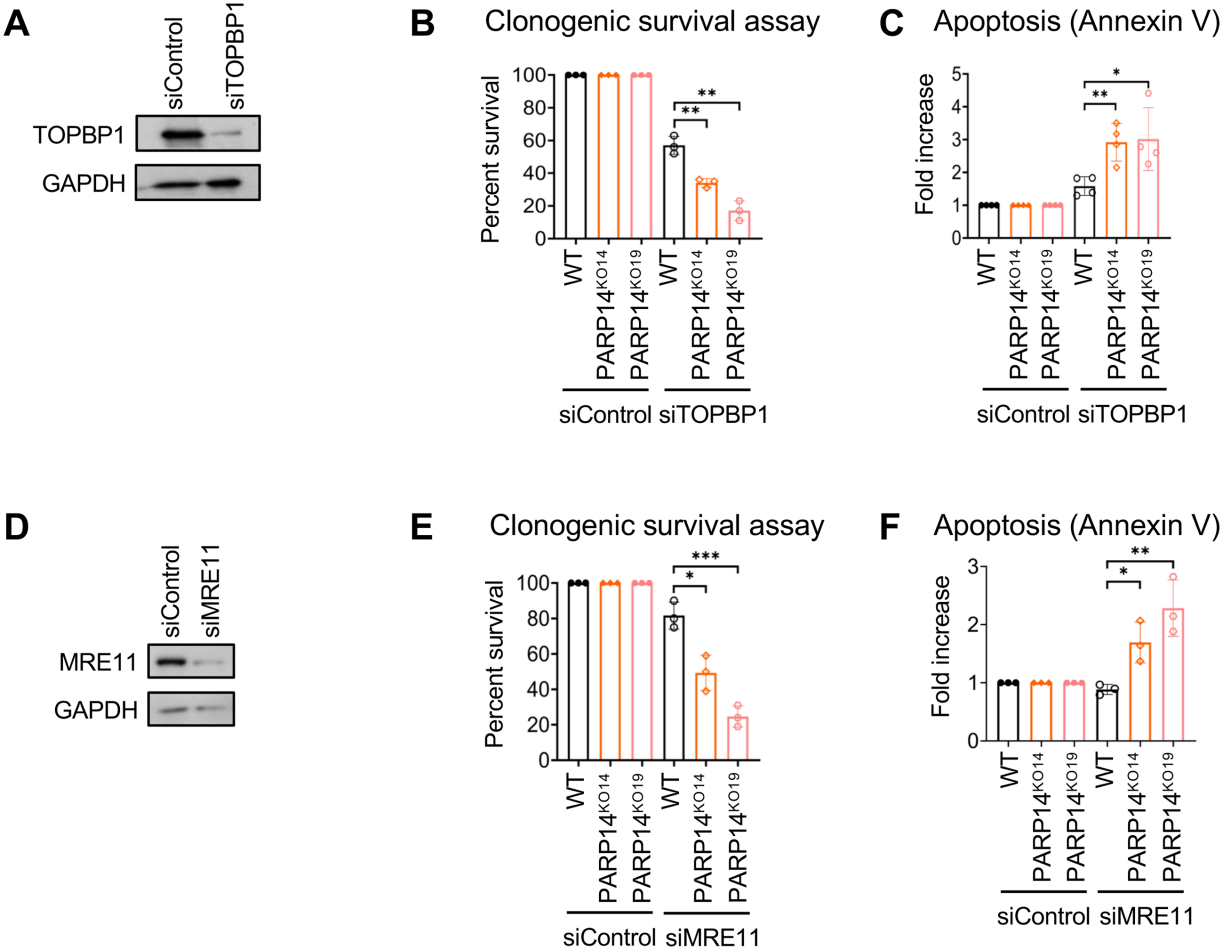
Reduced viability of PARP14-knockout cells upon inactivation of TOPBP1 or MRE11. (**A**) Western blot showing efficient siRNA-mediated downregulation of TOPBP1 in 8988T cells. (**B**) Clonogenic survival assays showing reduced survival of PARP14-knockout 8988T cells upon TOPBP1 knockdown. The average of three experiments is presented, with standard deviations shown as error bars. Asterisks indicate statistical significance. (**C**) Annexin V assays demonstrating increased apoptosis in PARP14-knockout 8988T cells upon TOPBP1 knockdown. The average of four experiments is presented, with standard deviations shown as error bars. Asterisks indicate statistical significance. (**D**) Western blot showing efficient siRNA-mediated downregulation of MRE11 in 8988T cells. (**E**) Clonogenic survival assays showing reduced survival of PARP14-knockout 8988T cells upon MRE11 knockdown. The average of three experiments is presented, with standard deviations shown as error bars. Asterisks indicate statistical significance. (**F**) Annexin V assays demonstrating increased apoptosis in PARP14-knockout 8988T cells upon MRE11 knockdown. The average of three experiments is presented, with standard deviations shown as error bars. Asterisks indicate statistical significance.

In addition to CHK1, multiple other components of the ATR-CHK1 pathway were among the top hits in our PARP14 synthetic lethality screen, including RPA3, TOPBP1, RAD9A and MRE11 (Figure 1C, E). TOPBP1 and MRE11, which is a member of the MRN complex, co-operate to activate ATR in response to replication stress (43–46). Thus, we decided to also validate these two candidates. Western blot experiments indicated that TOPBP1 can be efficiently depleted from 8988T cells (Figure 4A). Similar to observations made with the other top hits, knocking down TOPBP1 led to impaired colony formation in two different PARP14-knockout 8988T clones (Figure 4B). Moreover, TOPBP1 depletion significantly increased apoptosis in these cells (Figure 4C).

Finally, we also depleted MRE11 from 8988T cells (Figure 4D). MRE11 knockdown in two different PARP14-knockout clones resulted in reduced clonogenic survival (Figure 4E), and increased apoptosis (Figure 4F). These findings confirm that TOPBP1 and MRE11, upstream components of the ATR pathway, are required for viability of PARP14-deficient cells. Moreover, these findings further validate our CRISPR knockout screen.

### PARP14-knockout cells show hypersensitivity to pharmacological inhibition of the ATR-CHK1 pathway

**Figure 5.**
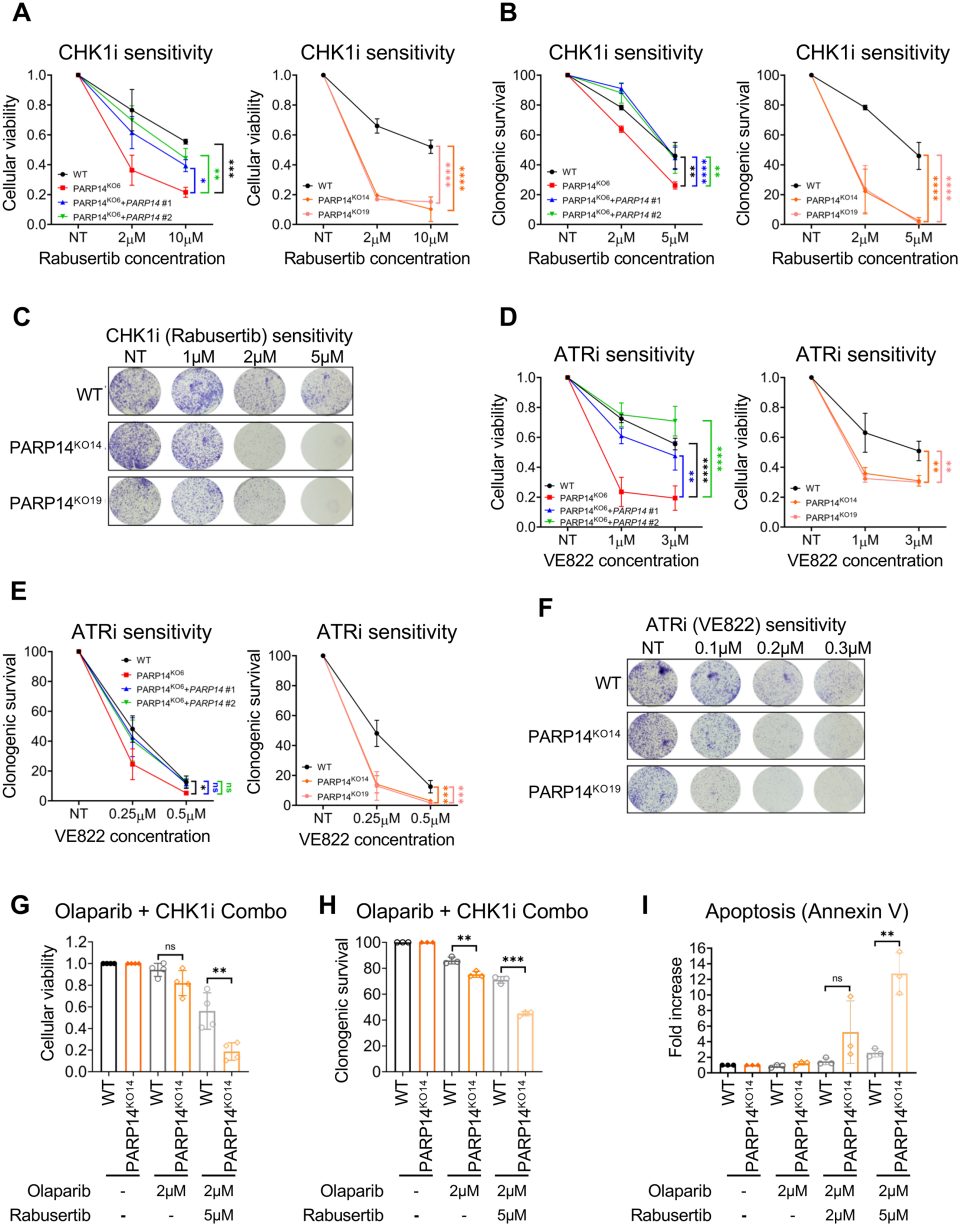
Loss of PARP14 sensitizes cells to inhibitors of the ATR-CHK1 pathway. (A, B) Increased sensitivity of 8988T PARP14-knockout cells to the CHK1 inhibitor rabusertib, in both cellular viability (**A**) and clonogenic (**B**) assays. Multiple knockout clones show the same phenotype. Re-expression of PARP14 in the knockout cells restores CHK1i resistance. The average of three experiments is presented, with standard deviations shown as error bars. Asterisks indicate statistical significance. (**C**) Crystal violet staining showing increased rabusertib sensitivity of 8988T PARP14-knockout cells. (D, E) Increased sensitivity of 8988T PARP14-knockout cells to the ATR inhibitor VE822, in both cellular viability (**D**) and clonogenic (**E**) assays. Multiple knockout clones show the same phenotype. Re-expression of PARP14 in the knockout cells restores ATRi resistance. The average of three experiments is presented, with standard deviations shown as error bars. Asterisks indicate statistical significance. (**F**) Crystal violet staining showing increased VE822 sensitivity of 8988T PARP14-knockout cells. (**G, H**) CHK1 inhibition potentiates the olaparib sensitivity of PARP14-knockout 8988T cells in both cellular viability (**G**) and clonogenic (**H**) assays. The average of three experiments is presented, with standard deviations shown as error bars. Asterisks indicate statistical significance. (**I**) Annexin V assays demonstrating increased apoptosis in PARP14-knockout 8988T cells upon concomitant treatment with CHK1 and PARP1 inhibitors. The average of three experiments is presented, with standard deviations shown as error bars. Asterisks indicate statistical significance.

Pharmacological inhibition of enzymatic activity is a key approach in personalized cancer therapy. Having observed that CHK1 depletion impairs cellular viability of PARP14-knockout cells, we wanted to confirm these observations using a pharmacological approach. Rabusertib (LY2603618) is a selective CHK1 inhibitor (CHK1i). To test sensitivity of PARP14-deficient cells to CHK1 inhibition, we measured cellular proliferation of PARP14-knockout cells treated with increasing concentrations of rabusertib. Cellular viability of all three PARP14-knockout clones was significantly reduced compared to wildtype control (Figure 5A). Re-expression of exogenous P*ARP14* cDNA in PARP14^KO6^ cell line restored cellular viability (Figure 5A). Similar results were obtained when using clonogenic survival assays (Figure 5B, C).

Since CHK1 is the key downstream factor in the ATR pathway (4,42), and multiple additional components of this pathway were top candidates in our PARP14 synthetic lethality screen (Figure 1C, E), we sought to investigate whether PARP14-knockout cells are also sensitive to ATR inhibitors (ATRi). To this end, we treated cells with VE822, a selective ATRi. All three PARP14-knockout clones demonstrated higher sensitivity to VE822, in both cellular viability (Figure 5D) and clonogenic survival (Figure 5E, F) assays. This sensitivity was suppressed upon re-expression of wildtype *PARP14* cDNA in the PARP14^KO6^ clone (Figure 5D, E). These findings show that PARP14-deficient cells are sensitive not only to genetic depletion of CHK1, but also to pharmacological inhibition of the ATR-CHK1 pathway.

We previously showed that PARP14 is involved in HR, and thus cells depleted of PARP14 by siRNA show slight sensitivity to the PARP1 inhibitor olaparib (29). We observed a similar trend for the PARP14-knockout 8988T cells in both cellular viability (Figure 5G) and clonogenic (Figure 5H) assays. However, co-treatment with the CHK1 inhibitor rabusertib increased the olaparib sensitivity of PARP14-knockout cells (Figure 5G, H). Moreover, co-treatment with rabursetib and olaparib increased apoptosis in PARP14-knockout cells compared to control cells (Figure 5I). These findings further attest to the importance of the PARP14 status as an important genetic determinant of the cellular response to cancer drugs targeting the DNA repair system.

### Altered replication fork dynamics underlie the synthetic lethality between PARP14 and the ATR pathway

**Figure 6.**
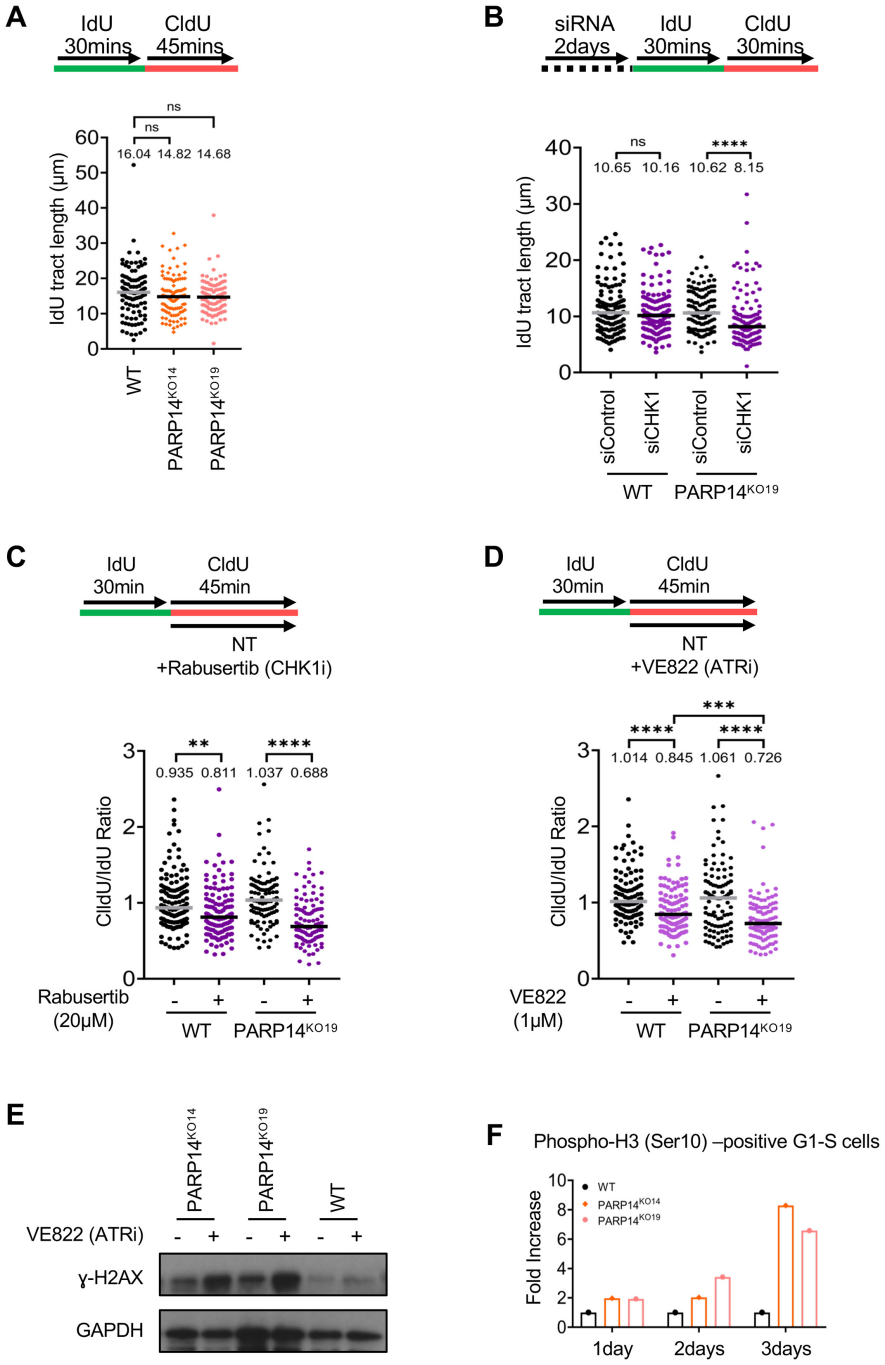
Impact of ATR-CHK1 pathway inhibition on replication dynamics of PARP14-knockout cells. (**A**) DNA fiber combing assay showing normal replication tract length in multiple PARP14-knockout clones. (**B**) Knockdown of CHK1 reduces replication tract length in PARP14-knockout 8988T cells. (**C, D**) Inhibition of CHK1 (**C**) or ATR (**D**) reduces replication tract length in PARP14-knockout 8988T cells. For all DNA fiber experiments (A–D), the median values are indicated for each sample, and the asterisks indicate statistical significance. At least 100 fibers were quantified. A schematic representation of the assay is shown at the top. (**E**) Western blot showing that H2AX phosphorylation is increased in PARP14-knockout cells upon treatment with 0.25 μM VE822 for 24 h. (**F**) Quantification of G1 and S cells with phosphorylated histone H3 at Ser10 as detected by flow cytometry. The fold increase in the difference of the percent of cells positive for pH3-Ser10 between ATRi-treated (0.25 μM VE822 for the indicated number of days) and untreated, normalized to wildtype is shown (*n* = 1). A graph presenting the number of cells in each category is shown in Supplementary Figure S4.

Upon replication stress, the ATR–CHK1 pathway promotes replication fork stability, preventing fork collapse and chromosome breakage (47–49). Thus, we investigated replication fork dynamics by employing the DNA fiber combing assay to measure the progress of individual replication forks, upon consecutive incubations with thymidine analogs IdU and CldU. Immunofluorescence microscopy-based detection of replication tracts indicated that, under normal growth conditions, loss of PARP14 does not affect replication tract length (Figure 6A). However, CHK1 depletion significantly reduced replication tract length in PARP14-knockout cells (Figure 6B). We further validated the knockdown studies by employing pharmaceutical inhibitors of the ATR1–CHK1 pathway. Similar to the knockdown studies, CHK1 inhibition in PARP14-knockout cells resulted in a stronger reduction in replication tract length in PARP14-knockout cells compared to control cells (Figure 6C). Similar results were observed for ATR inhibition (Figure 6D). These results indicate an increased necessity for ATR activation to maintain viability of PARP14-deficient cells, perhaps reflecting increased endogenous replication stress in these cells. In line with this, we observed increased γH2AX in PARP14-knockout cells, both under normal growth conditions and in particular upon ATR inhibition (Figure 6E), indicating that ATR-mediated fork protection suppresses accumulation of abnormal DNA structures in PARP14-deficient cells.

The decreased replication fork tracts in PARP14-deficient cells upon ATR inhibition suggest that replication is deficient in these cells, possibly because of fork arrest at endogenous lesions. The ATR pathway is also important for suppressing cell cycle progression in the presence of DNA damage, and its inhibition results in premature mitotic entry of cells with DNA damage (50,51). Histone H3 phosphorylation at Ser10 is a marker of chromosome condensation in mitosis. We observed an increase in G1-S cells positive for H3-Ser10 phosphorylation upon ATR inhibition in PARP14-knockout cells (Figure 6F, Supplementary Figure S4). Overall, these findings indicate that, upon concomitant loss of PARP14 and the ATR pathway, DNA replication is defective, and cells with incompletely replicated DNA enter mitosis resulting in mitotic catastrophe, thereby explaining the loss of viability.

## DISCUSSION

Genome-wide screens are powerful tools to investigate biological roles of the gene of interest in an unbiased manner. The PARP14 synthetic lethality screen described here identified a number of biological processes with which PARP14 activity has been previously associated. The most highly represented biological processes involved RNA metabolism, in line with previously published work indicating a role for PARP14 in RNA stability (23,26). In addition, this may also reflect a possible activity of PARP14 in directly binding RNA, as PARP14 contains two RRM domains, which are known to bind RNA (16). On the other hand, protein translation stress has been shown to induce DNA damage and replication stress, and activate the DNA damage response (52–54). Thus, it is possible that the prevalence of RNA metabolism in the list of top biological pathways showing synthetic lethality with PARP14, indicates a functional connection with the ATR pathway. Finally, biological processes highly enriched among the top hits which were previously associated with PARP14 functions include mitochondrial activity (21,27) and the DDR (29).

Understanding how the specific molecular make-up of the tumor modulates its response to therapy allows improved utilization of cancer drugs. This is relevant for both classic genotoxic chemotherapeutics such as DNA damaging compounds (e.g. cisplatin) and replication inhibitors (e.g. hydroxyurea), as well as for the new generation of chemical inhibitors such as those targeting the DDR, including PARP1 inhibitors and inhibitors of the ATR-CHK1 pathway. By employing a genome-wide CRISPR knockout screen aimed at unbiased identification of synthetic lethal interactors of PARP14, we found that the ATR-CHK1 pathway was essential for viability of PARP14-deficient cells, in multiple cell lines. PARP14-deficient cells were hypersensitive to both genetic depletion, and pharmacological inhibition of this pathway. Multiple components of the pathway were identified, including the upstream components TOPBP1, MRE11, RPA3 and RAD9A, and the downstream kinase CHK1. Although not a direct component of the ATR pathway, the DNA2 nuclease-helicase, another top hit we validated, has been found to participate in ATR activation under certain conditions, at least in yeast (41,55). These findings highlight an important role of PARP14 in the response to replication stress.

Mechanistically, we identified the control of DNA replication fork dynamics as a potential contributor to the synthetic lethality between the ATR-CHK1 pathway and PARP14. We show that DNA replication is compromised upon concomitant loss of PARP14 and the ATR pathway, and cells with incompletely replicated DNA undergo premature mitotic entry, thereby explaining the synthetic lethality observed.

The ATR-CHK1 pathway suppresses origin firing, thus its inhibition increases the number of replication forks and decreases the nucleotide pools in the cell, resulting in slower replication fork speed (6,47–49). In PARP14-deficient cells this slowing is accentuated, resulting in severe replication deficiency. We hypothesize that this occurs because of increased stalling of the replication forks at sites of DNA lesions, resulting in accumulation of abnormal DNA structures. However, it is also possible that this reflects a role of PARP14 in replication origin firing. Indeed, another top candidate from the synthetic lethality screen is CDC7 (Figure 1E), a kinase which regulates origin firing (56).

In conclusion, we identified an unexpected role for the ATR-CHK1 pathway in promoting cellular viability in the context of PARP14 deficiency. Our work indicates that the status of the *PARP14* gene in the tumor is an important determinant of the tumor response to DDR inhibitors, which are emerging as a powerful class of cancer drugs.

## DATA AVAILABILITY

All source data underlying each of the figures, including the values plotted in graphs, the exact *P*-values, and the uncropped blots are presented in Supplementary Table S4. The CRISPR screen datasets are available on BioGRID (57) (https://orcs.thebiogrid.org/dataset/dhoonmoon2020/) and DRYAD (https://doi.org/10.5061/dryad.p8cz8w9mj).

## Supplementary Material

gkaa508_Supplemental_Files
