# Supplementary material for: Genome-wide CRISPR synthetic lethality screen identifies a role for the ADP-ribosyltransferase PARP14 in DNA replication dynamics controlled by ATR
Source: Nucleic Acids Res. 2020 Jun 15;48(13):7252–64. doi: 10.1093/nar/gkaa508 (PMC7367200; doi:10.1093/nar/gkaa508)

SUPPLEMENTARY MATERIAL

LEGENDS TO SUPPLEMENTARY TABLES

Supplementary Table S1. List of all genes and gRNAs in the PARP14 synthetic lethality CRISPR screen ranked by RSA. The "Gene Rank RSA" tab lists all genes ranked by p-value as obtained from the RSA analyses. The "gRNA List" tab lists all gRNA sequences and indicates the read count for each of them.

Supplementary Table S2. List of all genes and gRNAs in the PARP14 synthetic lethality CRISPR screen ranked by MAGeCK. The "Gene Rank MAGeCK" tab lists all genes ranked by p-value as obtained from the MAGeCK analyses. The "gRNA List" tab lists all gRNA sequences and indicates the normalized read count for each of them as obtained from the MAGeCK analyses.

Supplementary Table S3. The top 25 Gene Ontology terms from the pathway analysis of the top 500 hits (ranked by RSA or MAGeCK, as indicated for each tab), listing the genes from each pathway which were hits.

Supplementary Table S4. The source data underlying each of the figure panels, including: the values plotted in graphs, the exact p-values, and the uncropped blots.

LEGENDS TO SUPPLEMENTARY FIGURES

Supplementary Figure S1. The script used for bioinformatic analysis of the screen using the RSA (**A**) and MAGeCK (**B**) algorithms.

Supplementary Figure S2. Bioinformatic analyses of the screen by RSA and MAGeCK yield similar results. **(A)** Pathway analysis showing the biological processes that were significantly enriched in the top 500 hits (genes lost in the PARP14^{KO6} cells compared to wildtype, ranked using MAGeCK). The top 25 Gene Ontology (GO) terms are shown. (**B**) Comparison of the ranking of top DNA damage response hits by RSA and MAGeCK.

Supplementary Figure S3. Impact of PARP14 knockout on cell cycle distribution. (**A**) PARP14^{KO6} 8988T cells show a similar cell cycle distribution as control wildtype cells. In addition, CHK1 knockdown does not

impact cell cycle distribution in wildtype compared to PARP14-knockout cells. (**B**) PARP14^{KO14} and PARP14^{KO19} 8988T cells show a similar cell cycle distribution as control wildtype cells.

Supplementary Figure S4. Graph showing the data plotted in Figure 6F, but presented here as the number of cells in each category.

Supplementary Figure S1

A RSA analysis

#Get fastq Files
#Unzip Files

>> module load python/2.7.6
>> for file in *.fastq; do filename=`echo $file | cut -d "." -f 1`; python count_spacers4.py -o ${filename}_out.csv -i Calabrese_library_sequences_header.csv -f $file; done

#Check Statistics.txt to see library representation

#Get Output files and do calculations in excel
#Normalize the amount of reads to the total amount of reads for that sample
#Find the score of each guide
=LOG(ExperimentalNormalizedValue/ControlNormalizedValue,2)
#Save new file with just sgRNA.Target.Sequence and Score
#Use R to merge sequences with Gene_ID

>> module load R
>> R
>> Score <- read.csv(file = "Score.csv", stringsAsFactors = F)
>> Ref <- read.csv(file = "Calabrese_Reference.csv", stringsAsFactors = F)
>> merge(x = Ref, y = Score, by "sgRNA.Target.Sequence") -> GeneIDFile
>> write.csv (GeneIDFile, file = "GeneIDFile.csv")

#Run RSA

>> tar -xvf RSA.1.9.tar
>> chmod -x+w RSA.1.9.tar
>> module load python/2.7.9
>> for file in *.csv; do filename=`echo $file | cut -d "." -f 1`; python RSA/RSA.py -b -r -o ${filename}_out.txt $file; done

#Use R to add Gene Names

>> module load R
>> R
>> RSA_out <- read.csv (file = "RSA_out.csv", stringsAsFactors = F)
>> Symbol <- read.csv(file = "Symbols.csv", stringsAsFactors = F)
>> merge (x = Symbol, y = RSA_out, by "Gene_ID", all.y = T) -> RSA_Symbol
>> RSA_Symbol$Gene_ID <- as.character(RSA_Symbol$Gene_ID)
>> RSA_Symbol_InOrder <- RSA_Symbol[order(RSA_Symbol$LogP, RSA_Symbol$Gene_ID, RSA_Symbol$Score),]
>> write.csv (RSA_Symbol_InOrder, file = "RSA_Symbol_InOrder.csv")

B MAGeCK analysis

get fastq Files
unzip files

obtain library file – Brunello genome-wide library developed by the Broad Institute, file obtained from MAGeCK sourceforge - broadgpp-brunello-library-corrected.txt.zip
unzip library file

>> module load mageck
>> mageck count -l broadgpp-brunello-library-corrected.txt -n screen --sample-label PARP14KO,CTRL --fastq PARP14KO.fastq CTRL.fastq
>> mageck test screen.count.txt -t PARP14 -c CTRL screen

Supplementary Figure S2

A

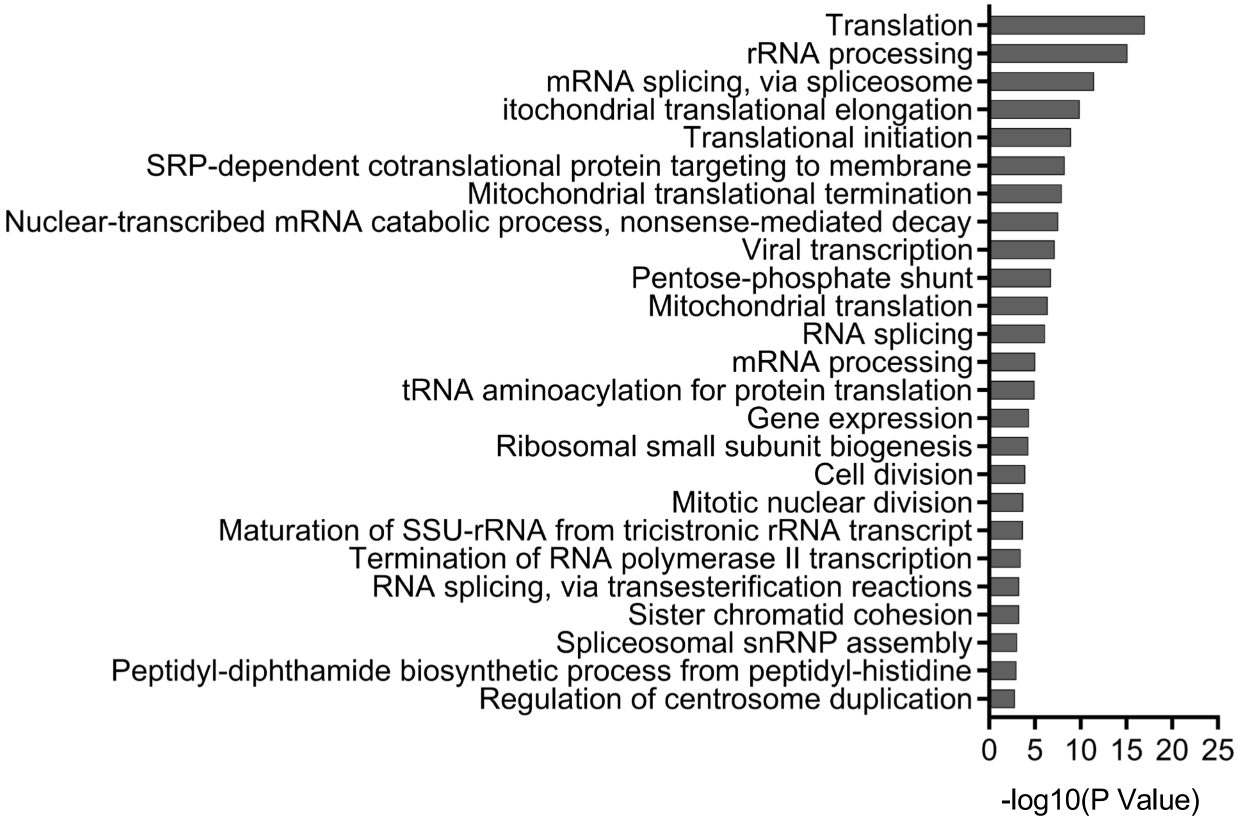

B

Gene Name	Rank using RSA	Rank using MAGeCK
RPA3	13	758
**CHK1**	**17**	**13**
CDC7	36	20
XRCC3	82	233
Fen1	116	166
RAD51C	121	599
CDC23	177	435
**TOPBP1**	**264**	**361**
**DNA2**	**428**	**540**
RAD9A	429	930
**MRE11**	**460**	**593**
XRCC5	612	1080

Supplementary Figure S3

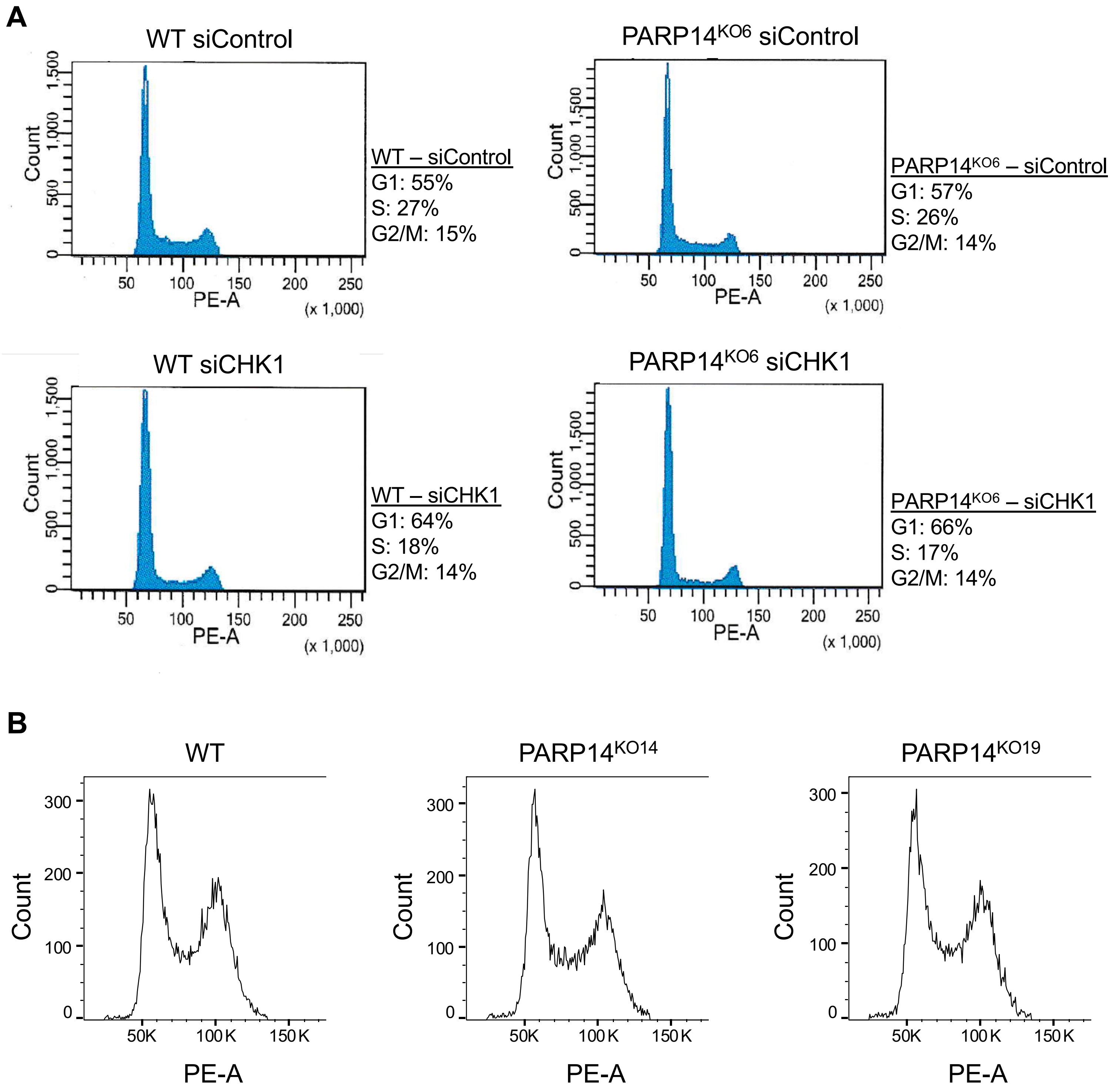

Supplementary Figure S4

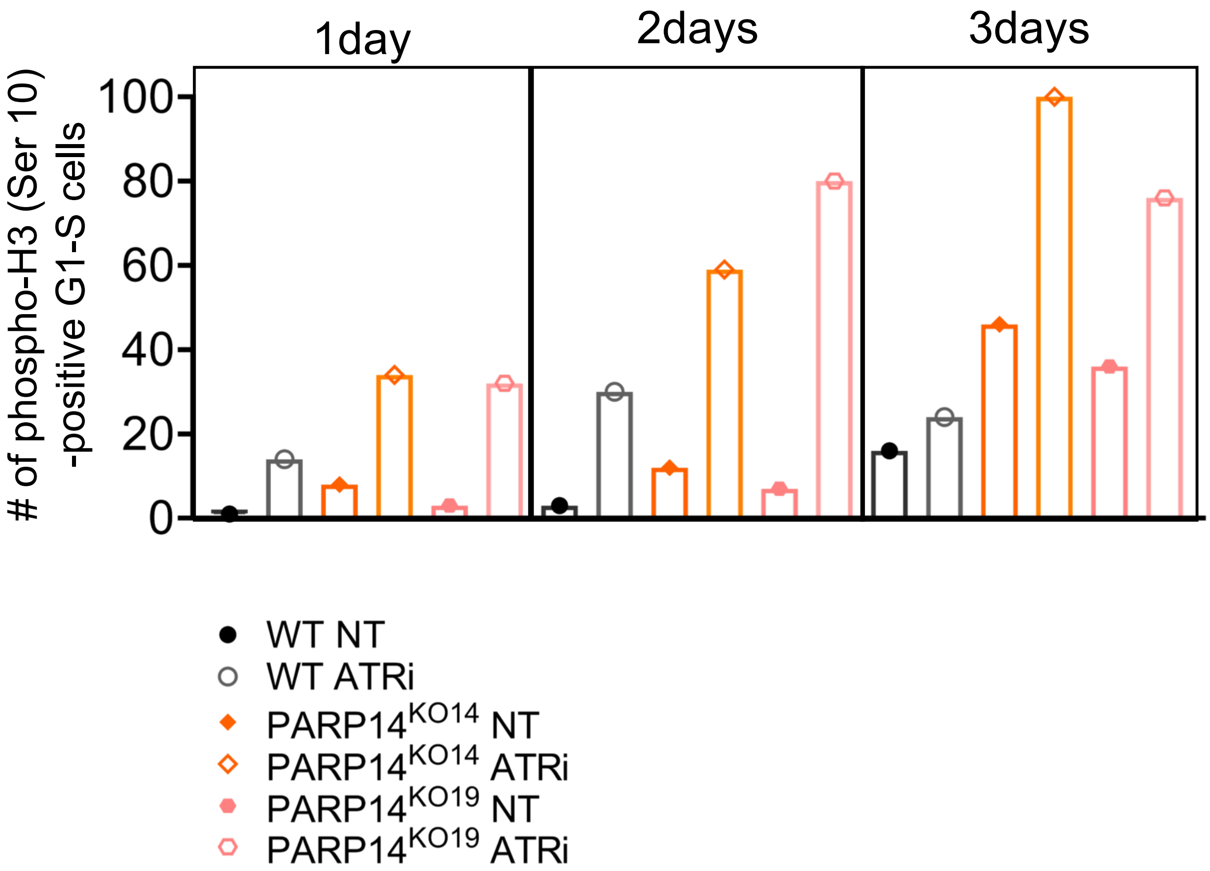

Supplement: gkaa508_Supplemental_Files [file gkaa508_supplemental_files.zip › Supplementary Material.pdf]
